# Acupuncture for hypertension with insomnia: Study protocol for a randomized, sham-controlled, subject-and-assessor-blinded trial

**DOI:** 10.3389/fpsyt.2022.1087706

**Published:** 2022-12-22

**Authors:** Xiaoqiu Wang, Pei Wang, Chengyong Liu, Shan Qin, Qingyun Wan, Shuting Luo, Wenzhong Wu

**Affiliations:** Jiangsu Province Hospital of Chinese Medicine, Affiliated Hospital of Nanjing University of Chinese Medicine, Nanjing, China

**Keywords:** acupuncture, insomnia, hypertension, randomized controlled trial, study protocol

## Abstract

**Background:**

Previous studies show that insomnia and hypertension are closely related. Currently, intervention for hypertension with insomnia has become a research hotspot. Acupuncture, as a representative non-pharmaceutical therapy of traditional Chinese medicine (TCM), has been widely used in improving insomnia and hypertension. However, there are few clinical studies on acupuncture for hypertension with insomnia.

**Methods:**

A single-center, subject-and-assessor-blind, randomized, sham-controlled trial has been designed for a study to be conducted in Jiangsu Province Hospital of Chinese Medicine. Sixty eligible patients will be randomly assigned to the treatment group and the control group in a 1:1 ratio. The treatment group will receive acupuncture treatment, while the control group will receive sham acupuncture treatment. Both groups will be treated three times per week for 4 weeks. Data will be collected at baseline and after 4 weeks of treatment and analyzed by using SPSS 25.0. The primary outcome measures are sleep parameters of portable polysomnography before and after treatment. Secondary outcomes are Pittsburgh Sleep Quality Index, Insomnia Severity Index, home blood pressure, and heart rate variability.

**Discussion:**

This study aims to evaluate the efficacy of acupuncture using the portable polysomnography combined with sleep scales, and analyze heart rate variability to preliminarily explore the underlying mechanism of acupuncture on hypertension with insomnia. The trail, if proven to be effective, will provide strong scientific evidence to support acupuncture is effective to manage patients for hypertension with insomnia.

**Clinical trial registration:**

ChiCTR2200059161.

## Introduction

Insomnia is one of the most prevalently encountered sleep disorder characterized by perceived difficulty in falling asleep and/or maintaining sleep, leading to unsatisfactory sleep (1). It has been reported that the prevalence of insomnia disorder in the general population has reached 10%−15% in China (2). Particularly, under the influence of factors such as the epidemic of coronavirus disease 2019 (COVID-19), the incidence rate is still ascending (3). Insomnia not only seriously harms patients' physical and mental health, but also increases the social medical burden (4). Studies have shown that insomnia can increase the risk of depression, dementia, hypertension, diabetes, tumors and other diseases (5–8). In particular, the relationship between insomnia and hypertension has attracted more and more attention from researchers over the last few decades. Hypertension, the most common chronic disease, is the main cause of death in patients with cardiovascular and cerebrovascular diseases. In China, 2.33 million people die from cardiovascular and cerebrovascular diseases caused by hypertension every year (9). The awareness rate, treatment rate and control rate of hypertension were 46.9, 40.7, and 15.3%, respectively, which are far lower than developed countries (10). Therefore, it is crucial to seek a positive and stable antihypertensive strategy for preventing cardiovascular and cerebrovascular diseases in China.

There are many factors influencing hypertension (11), such as smoking, diabetes, dyslipidemia, obesity, and unhealthy diet. Currently, it is clinically found that insomnia disorder is a common complaint of patients with hypertension, and insomnia and hypertension often co-exist. The findings (12) have suggested that the proportion of insomnia in hypertensive patients is 19%−47.9% compared with normal blood pressure, and the incidence of hypertension in insomnia patients is 21.4%−50.2% compared with normal sleepers. Numerous meta-analyses (7, 13, 14) have reported that insomnia is an important risk factor for hypertension. Jarrin et al. (13) summarized the potential mechanisms related to insomnia and hypertension, suggesting that insomnia may cause persistent hypertension through (but are not limited to) the activation of the autonomic nervous system (e.g., hyperactive sympathetic, lower parasympathetic), the renin-angiotensin-aldosterone system, the hypothalamic-pituitary-adrenal axis, and the immune system. In the past, due to the lack of public awareness of the relationship between insomnia and hypertension, most patients with hypertension and insomnia did not receive formal and active insomnia intervention in clinical practice, which is one of the reasons that blood pressure in patients with hypertension is difficult to control. At present, as the relationship between insomnia and high blood pressure is increasingly becoming a new focus of attention, many reports (15–17) have manifested that the combination with insomnia intervention on the basis of blood pressure reduction can contribute to controlling blood pressure and correcting blood pressure rhythm while ameliorating insomnia symptoms. However, benzodiazepines, the most commonly used in clinical treatment of insomnia, exist well-known side effects such as next-day hangover and drug dependence, which are not easily accepted by patients (18). The guideline-recommended cognitive behavioral therapy for insomnia (CBT-I) is difficult to implement clinically owing to its professionalism, long time-consuming, and poor patient compliance (19). Hence, it is critical to find safe and effective non-pharmaceutical therapy to further optimize the treatment mode of hypertension complicated by insomnia.

Acupuncture, as the most representative non-pharmaceutical therapy of TCM, has been widely applied to treat a variety of diseases ascribe to its unique advantages such as simplicity, safety, and no side effects. Now, acupuncture treatment of insomnia has been recommended by the “*Guidelines for the Diagnosis and Treatment of Insomnia in China*” (2). The meta-analysis (20) has revealed that acupuncture is effective in insomnia, and it is not a placebo effect. A small number of domestic studies have shown that acupuncture can improve the sleep quality of patients with hypertensive stroke, and the improvement of sleep quality can assist in regulating the early morning blood pressure of patients (21, 22). However, there are completely few clinical studies on the intervention of acupuncture on hypertension with insomnia, and it is still inconclusive whether acupuncture can improve insomnia and regulate blood pressure at the same time. In addition, the current researches have problems such as sleep outcome indicators mainly relying on scales and lack of objective evaluation. However, polysomnography (PSG), a “gold standard” assessment tool for the diagnosis of sleep disorders, is cumbersome to operate due to its large size and multiple leads, which not only affects the natural sleep state and fails to describe the real sleep state, but also lacks long-term monitoring conditions, causing it inconvenient for clinical practice (23). Compared with PSG, portable polysomnography (PPSG) has the superiorities of simple operation and home monitoring, small first-night effect, strong endurance and so on. Artificial Intelligence (AI) algorithm was used to evaluate sleep structure and sleep quality in detail, analyze and mark manual/automatic sleep-related events, judge and count micro-awakenings, and quickly identify sleep characteristic waveform, with an accuracy of 95% (24). The validity of PPSG in assessing Sleep has been recognized in several researches and meets the standards of America Academy of Sleep Medicine (AASM) (25).

Sympathetic nervous system hyperactivity is often considered to be one of the mechanisms for the comorbidity of insomnia and hypertension (26, 27). The hyperarousal hypothesis holds that chronic insomnia is a manifestation of physiological hyperarousal, during which insomnia patients have a persistent increase in sympathetic excitation. And sympathetic nerve overexcitation will constrict blood vessels and increase blood flow resistance, thereby increasing blood pressure and increasing the risk of hypertension (28). Moreover, the autonomic nervous system is involved in the regulation of the circadian rhythm of blood pressure, and rhythm disorders of the autonomic nervous system can cause blood pressure rhythm disturbance, resulting in non-dipper or even anti-dipper blood pressure rhythms (29, 30). Heart rate variability (HRV) is a non-invasive quantitative indicator that can effectively monitor autonomic nerve regulation in clinical practice, and can reflect individual sympathetic and parasympathetic nervous function states (31, 32). Therefore, the change of heart rate variability is the underlying mechanism of acupuncture intervention on the comorbidity of hypertension and insomnia.

Based on the above, we design a randomized, sham-controlled trial that the main purpose is to explore the effects of acupuncture on objective sleep and blood pressure of hypertensive patients with insomnia by using PPSG combined with sleep scales and the secondary objective is to investigate the mechanism of acupuncture treatment in the comorbidity of hypertension and insomnia by HRV data collected by single-lead portable electrocardiograph (ECG).

## Methods/design

### Design

**Figure 1:**
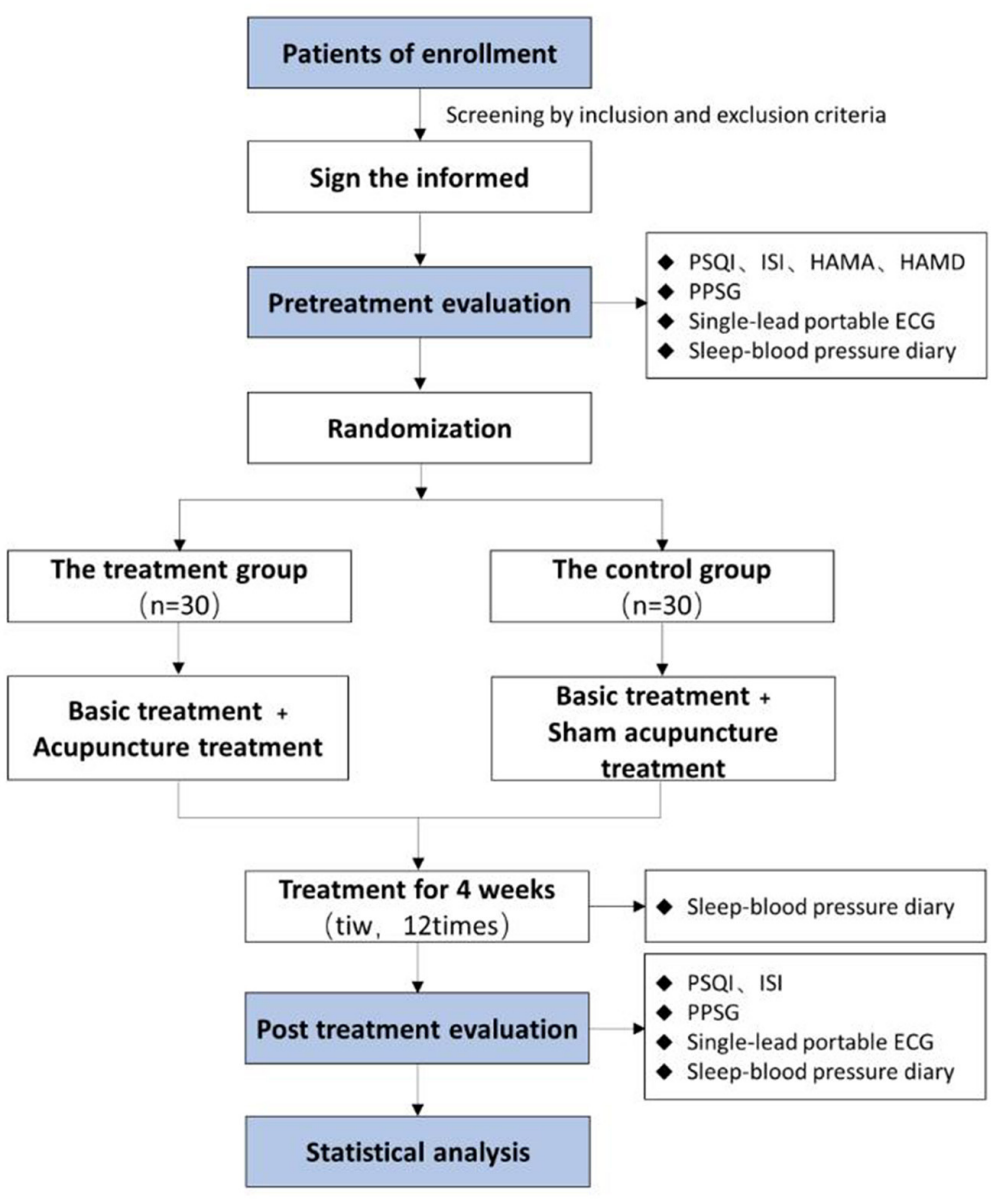
Flow diagram of the trial.

A single-center, subject-and-assessor-blind, randomized, sham-controlled trial will be performed at Jiangsu Province Hospital of Chinese Medicine (Nanjing, China). A total of 60 participants will be randomly assigned to the treatment group (acupuncture group) and the control group (sham acupuncture group) in a 1:1 allocation ratio. Both groups will be treated three times per week for 4 weeks (12 sessions totally). The clinical trial conforms to Standard Protocol Items: Recommendations for Interventional Trials (SPIRIT) 2013 statement (33) as well as the Standards for Reporting Interventions in Controlled Trials of Acupuncture (STRICTA) (34). The study procedure and details are summarized in Figure 1 and Table 1.

**Table 1:** Schedule of enrolment, interventions, and assessments.

**Time point**	**Study period**			
	**Baseline**	**Intervention**		**Post-** **treatment**
	−**1 week**	**Week 0**	**Week 4**	
**Enrolment**				
Eligibility screen	•			
Informed consent	•			
Medical history	•			
Physical examination	•			•
Random allocation	•			•
**Intervention**				
Acupuncture group (*n* = 30)		12 sessions of acupuncture in acupoints		
Sham acupuncture group (*n* = 30)		12 sessions of acupuncture with slight skin penetration		
Assessments				•
PSQI	•			•
ISI	•			•
HAMA	•			
HAMD	•			
Sleep-blood pressure diary	•	•	•	•
PPSG	•			•
Single-lead portable ECG	•			•
**Others**				
Success of blinding				•
Adverse events check		•	•	

PSQI, Pittsburgh Sleep Quality Index; ISI, Insomnia Severity Index; HAMA, Hamilton Anxiety Scale; HAMD, Hamilton Depression Rating Scale; PPSG, portable polysomnography; ECG, electrocardiograph.

### Patients

#### Recruitment strategies

This study will recruit patients of hypertension with insomnia through three approaches: (1) Regularly visit the Acupuncture and Moxibustion Rehabilitation Department, Physical Examination Center, and Hypertension Research Institute of Jiangsu Provincial Hospital of Traditional Chinese Medicine to collect cases; (2) Regularly distribute brochures in the halls of Jiangsu Provincial Hospital of Chinese Medicine or advertise on the screen to recruit qualified subjects; (3) Adopt WeChat platform to push articles to all the masses for recruitment.

#### Inclusion criteria

Patients meeting all of the following criteria will be enrolled in the study.

1. No gender restriction, from 18 to 70 years old;
2. Meeting the International Classification of Sleep Disorders-Third Edition (ICSD-3) diagnostic criteria for insomnia;
3. 8 ≤ Insomnia Severity Index (ISI) score ≤ 22;
4. Meeting the Chinese Guidelines for the Prevention and Treatment of Hypertension (2018 Revised Edition) diagnostic criteria of primary hypertension (35);
5. The cardiovascular risk stratification is low to medium risk (35), the blood pressure control is relatively stable, and the types of hypertension drugs taken are Calcium Channel Clocker (CCB)+ Angiotensin-Converting Enzyme Inhibitors (ACEI)/Angiotensin Receptor Blockers (ARB), but no more than two types;
6. Voluntarily participate in this trial and sign the informed consent.

#### Exclusion criteria

Patients meeting any of the following criteria will be excluded from the study:

1. Patients with serious physical diseases, such as heart failure, myocardial infarction, cerebral infarction, malignant tumor, etc.;
2. Hamilton Anxiety Scale (HAMA) score ≥2 or Hamilton Depression Rating Scale (HAMD) score ≥18;
3. Caffeine, alcohol or psychotropic substances dependence;
4. Other types of sleep disorders such as sleep apnea and restless legs syndrome;
5. Night shift workers;
6. Stop insomnia-related interventions (including traditional Chinese medicine, acupuncture, massage, and western medicine) ≤ 2 weeks;
7. Change of antihypertensive plan within the last 1 month;
8. Pregnant and lactating women;
9. Patients are participating in other trials.

#### Drop-out criteria

1. Patients who refused to cooperate with the test and diagnostic errors after enrollment were considered as excluded cases;
2. During the study period, patients dropped out, did not complete the whole course of treatment, and poor compliance were considered as abscission cases.
3. Patients who could not tolerate acupuncture, had serious adverse events during treatment, or had serious complications caused by other diseases would terminate the trial.

### Intervention

#### Initial therapy

Both groups will obtain unified sleep hygiene education and hypertension health education by means of pushing articles through the WeChat platform. All patients will be allowed to routinely take their hypertension medication.

#### The treatment group

The treatment group will be given acupuncture treatment at DU20 (Baihui), EX-HN3 (Yintang), HT7 (Shenmen), SP6 (Sanyinjiao), LR3 (Taichong), and KI3 (Taixi), and the above acupoints will be strictly in accordance with *National Standard of China: Meridian Points* (GB12346–90). The subjects will be placed in the supine position and the physician will select 0.30 mm × 40 mm disposable sterile acupuncture needles (Suzhou Medical Products Factory Co., Ltd.). The angle and depth of needling will be based on the standard of each acupoint. The acupuncture needles will be inserted into the acupoints and lifted and twisted to achieve the state of *deqi*. The low-frequency pulse electro-acupuncture therapeutic apparatus (XS-998B04; Nanjing Xiaosong Medical Instrument Research Institute Co., Ltd., Nanjing, China) will be used to connect the needle handles at DU20 and EX-HN3, and the electric stimulation will be applied with a 2 Hz frequency and continuous wave type at an intensity that the subject can notice but feel comfortable with. After continuous stimulation for 30 min, the points will be compressed with a sterilized cotton ball to prevent bleeding upon needles withdrawal. Participants will accept acupuncture treatment three times a week for 4 weeks (12 sessions totally). All acupuncture procedures will be performed by the same acupuncturist.

#### The control group

The sham-acupuncture method of shallow acupuncture at non-disease-related points will be applied to the control group. LI10 (Shousanli), LI14 (Binao), ST32 (Futu), BL58 (Feiyang) acupoints will be selected, and the acupoints will be strictly in accordance with National Standard of China: Meridian Points (GB12346–90). The subjects will be placed in the supine position and the physician will select 0.30 mm × 25 mm disposable sterile acupuncture needles (Suzhou Medical Products Factory Co., Ltd.). After routine disinfection, the needles will be inserted only for 1–2 mm and avoid manual stimulation and the sensation of *deqi* in order to minimize placebo effect. The low-frequency pulse electroacupuncture therapeutic apparatus (same as the treatment group) will be placed beside the patient, and the needle handle will be connected at LI10 and ST32 acupoints on one side, the apparatus will turned on, but the stimulation intensity button will not turned on, and the needle will removed after 30 min. Participants will accept sham-acupuncture treatment three times a week for 4 weeks (12 sessions totally). At the end of the study, the same treatment as the treatment group was received. All acupuncture procedures will be performed by the same acupuncturist.

### Outcome measures

#### Primary outcome measures

PPSG is one of the sleep monitoring methods with the advantages of supporting home environment monitoring and avoiding the influence of the first night effect, which can be used for objective diagnosis of insomnia, treatment evaluation and differential diagnosis of sleep diseases. In this study, Anbaolan wearable portable sleep recorder [202070644; Embla (Beijing) Medical Equipment Co., Ltd., Beijing, China] was applied to sleep monitoring. The device records electroencephalogram (EEG), electrooculogram (EOG) and electromyogram (EMG) signals through a set of head-mounted sensors, monitors blood oxygen with finger pulse oxygen, and uses AI intelligent algorithms to evaluate sleep structure in detail, including wakefulness (W) and rapid eye movement sleep (REM) sleep period), N1 stage sleep, N2 stage sleep, N3 stage sleep, and then the sleep quality evaluation indicators such as sleep efficiency (SE), sleep awakening times (SA) and total sleep time (TST) can be obtained, which can provide real-time and objective sleep evaluation indices for individuals. Sleep parameters of PPSG will be collected one night and evaluated before treatment and after 4 weeks of treatment.

#### Secondary outcome measures

Pittsburgh Sleep Quality Index (PSQI) is a questionnaire to assess the sleep quality of individuals. The total score of PSQI is 0–21 and the score is inversely proportional to the sleep quality, namely, the higher the PSQI score, the worse the sleep quality. PSQI > 7 was used as the reference threshold for judging sleep quality problems. PSQI will be evaluated before treatment and after 4 weeks of treatment.

ISI questionnaire is designed to evaluate severity of insomnia through 7 self-assessment items corresponding to the symptoms of insomnia. In the present study, 8 ≤ ISI ≤ 22 will be taken as the inclusion criterion. ISI score will be measured before treatment and after 4 weeks of treatment.

HAMA is created to evaluate patients' anxiety symptoms. There are 14 items scored by 5-point scale from 0 to 4. According to the data provided by the China Scale Collaboration Group, a total score <7 indicates no anxiety symptoms, a score of 7–13 indicates possible anxiety, a score of 14–20 indicates definite anxiety, a score of 21–28 indicates definite anxiety, and a total score ≥29 indicates severe anxiety. The depressive symptoms will be assessed using Hamilton Depression Rating Scale (HAMD) drawn with 24 items. The scoring criteria are as follows: a total score of <8 indicates normal, a total score of 8 to 17 indicates possible depression, a total score of 18–24 indicates depression, and a total score of >24 indicates severe depression. The above two scales are other-report questionnaires, which will be evaluated before enrollment to exclude individual with severe mental illness. HAMA score ≥29 or HAMD score ≥18 will be considered as one of the exclusion criteria in this study.

Sleep diary, a standard subjective assessment tool for insomnia, is used to observe the severity of insomnia in patients and monitor sleep improvement during treatment, which can capture sleep information such as the bedtime at the previous night, sleep latency, waking time in the morning, get-up time, the number and duration of wake times after sleep onset, total sleep time, in-bed time, and the use of sedative hypnotics. At the same time, blood pressure records will be added to the back of the sleep diary. The blood pressure monitoring method in this study adopts home blood pressure monitoring. Participants are required to bring their own Omron electronic blood pressure monitors at home, and the patients are instructed to record the systolic and diastolic blood pressure at 8 a.m. and 20 p.m. per day. During this study, participants will be allowed to use sedative-hypnotic drugs, but it is necessary to emphasize the recording of patients' daily sleeping pill use through a sleep diary, and the use of sleeping pills throughout the trial period will evaluated by calculating a weekly sleeping pill score as part of the efficacy analysis. The participants self-reported sleep-blood pressure diary will be collected from 1 week before treatment to the end of the trial.

Portable dynamic ECG recorder can collect and analyze total heart rate, mean heart rate, minimum heart rate, maximum heart rate, maximum RR interval, suprventricular beats, ventricular beats, heart rate variability and other parameters. In this study, the HRV indicators of participants will be collected by using the single-lead portable ECG recorder (JiangSu Registration No. 20182210915) produced by Nanjing Fengsheng Yongkang Software Technology Co., Ltd. The study mainly collected the heart rate of patients during sleep and analyzed the HRV. HRV usually denoted by the variance in the inter-beat interval or distance between two consecutive R waves, referred to as the R–R interval. We mainly analyzed the time-domain parameters of HRV, including the standard deviation of mean value between RR intervals (SDNN), root mean deviation of difference between adjacent RR intervals (RMSSD) and mean deviation of RR intervals (SDANN), and the percentage of the difference between adjacent NN >50 ms in the total number of sinus heartbeats (PNN50). The monitoring time is the same as PPSG. The two groups of patients were evaluated before treatment and at the end of treatment.

### Sample size

The calculation of sample size will be based on the change of ISI scores. According to the results of existing published literature (36), the overall standard deviation of ISI scores in patients of hypertension with insomnia was 5.5, ISI scores decreased by 3.3 after treatment, α = 0.05, β = 0.10, and *N* ≈ 24 cases were calculated. Considering the factors of contamination, non-compliance and shedding during the test, the shedding rate was determined to be 20%, so there were 30 cases in the treatment group and 30 cases in the control group. For comprehensive consideration, a total of 60 samples will be required for this study.

### Randomization and allocation concealment

After screening by inclusion and exclusion criteria, eligible participants will be divided into the treatment group and the control group according to the ratio of 1:1 by block random method, with 30 cases in each group. Random sequences will be generated using SPSS 25.0 software (IBM, Armonk, NY, USA) by researchers who are not involved in the implementation and statistical analysis of the experiment.

### Blinding

Grouping results will be kept confidential to participants, evaluators and statisticians. All participants will be asked to indicate whether they have received acupuncture or sham acupuncture within 5 min of treatment to assess blindness. Because the acupuncturist is required to perform acupuncture interventions, the grouping results will not be kept secret from the acupuncturist, the acupuncturist will not be able to evaluate the intervention results, and the evaluator will not be allowed to discuss the treatment.

### Informed consent

Participants will be informed of the details of the study, including the purpose of the study, potential benefits and risks, other available treatment options, and participant rights and obligations. Participants will be included in the study after signing written informed consent. If participants drop out of the trial, relevant data will be retained for final analysis.

### Safety monitoring

Adverse events (AEs) during treatment will recorded in a timely manner, including local hematoma, dizziness, and stagnation of acupuncture. Any serious AEs associated with the trial will be immediately reported to the main researchers, and we will determine its cause, analyze its correlation with acupuncture, and treat symptomatically.

### Data collection and management

Data from subjects was collected using the case report form (CRF) designed for this study and backed up into a database based on observed indicators. The Archives of Jiangsu Provincial Hospital of Traditional Chinese Medicine will keep these data for more than 10 years, during which time only members of the research team have access to these data.

### Statistical analysis

In this study, all statistical analyses will be conducted by an independent statistician using SPSS 25.0 software (IBM, Armonk, NY, USA). The measured data will be expressed as mean ± standard deviation, and the count data will be expressed by the ratio or composition ratio. For comparing measured data between the two groups, first normal analysis will be performed. A *t*-test will be used for the measured data conforming to normal and homogeneity of variance, and a nonparametric rank sum test will be used for non-normally distributed measured data. The data will be counted by the Chi-Square test or Fisher's exact test. *P* values ≤ 0.05 will be considered statistically significant.

### Quality control

To guarantee the quality of the trials, all participants, including acupuncturists, evaluators, and statisticians, will receive unified training. Acupuncturists with a master's degree in medicine, licensed physicians and clinical experience must strictly implement standardized intervention procedures. To familiarize the treatment options, all physician assistants are required to undergo a one-day professional training. To ensure consistency across the different participants, we will develop a set of clinical management practices. The above can ensure the feasibility and safety of clinical research.

### Trial status

The current protocol version is 2.0 as of 9 December 2021. The randomization began (recruitment) on 1 June 2022. Recruitment is expected to end in late 2022.

## Discussion

This study, a single-center, subject-and-assessor-blind, randomized, sham-controlled trial, aims to evaluate the efficacy of acupuncture using the PPSG combined with sleep scales, and to analyze HRV-related data to preliminarily explore the underlying mechanism of acupuncture in patients of hypertension with insomnia.

Insomnia is closely related to hypertension, which often exists in the form of comorbidity, seriously affecting patients' quality of life, physical and mental health, and increasing social medical burden. Acupuncture is widely used owing to its unique merits of simplicity, safety, and few side effects, which has potential advantages in the treatment of hypertension and insomnia (20, 37–39). However, there are few high-quality clinical studies on the intervention of acupuncture on hypertension with insomnia, and the following problems are common. First of all, the evaluation indicators of sleep are mainly based on subjective scales. Therefore, PPSG is first applied to the trail to assess objective sleep parameters. Secondly, the types of hypertension medication have been not clearly specified and limited in most studies. But researches have shown that beta-blockers such as metoprolol may reduce nocturnal melatonin production by blocking 5-HT, thus leading to increased number of awakenings, prolonged sleep latency and decreased sleep efficiency (40, 41). And diuretics give rise to increased number of nocturia and increased number of awakenings, which further leads to insomnia. Hence, this study will restrict the types of hypertension drugs, and the included patients will be only permitted to taken CCB+ACEI/ARB, so as to minimize the impact of hypertension drugs on sleep.

Thirdly, excessive activation of the sympathetic nervous system may be one of the mechanisms of the comorbidity of insomnia and hypertension (26, 27). HRV is a biological indicator reflecting the functional status of human sympathetic and parasympathetic nerves (41). Previous studies have shown that the nighttime HRV of insomnia patients is lower than that of healthy people (42), accompanied by increased heart rate, elevated blood pressure and other manifestations of enhanced sympathetic response (26, 43, 44). Meanwhile, elevated sympathetic activity is pivotal in the pathogenesis of hypertension (45). HRV parameters showed a strong correlation with hypertension, especially the time-domain index of HRV showed a significant negative correlation with hypertension (46). Lan et al. (47) used six HRV parameters to predict hypertension, and found that SDNN (one of the time-domain indicators) had the best predictive ability. Therefore, HRV, especially time-domain indicators, may be a potential marker reflecting the increased risk of hypertension in patients with insomnia. However, there is no research to explore the mechanism of acupuncture in the treatment of hypertension with insomnia, so this study used the change of HRV to initially explore the potential mechanism of acupuncture on the comorbidity of hypertension and insomnia.

Finally, in order to eliminate the placebo effect of acupuncture treatment as much as possible, sham acupuncture was used as a control in this study to reduce bias. According to the theory of traditional Chinese medicine, the main mechanisms of insomnia are Yin and Yang disorder, spirit falling to be nourished, and absence of mind. Hence, we choose DU20 (Baihui) and EX-HN3 (Yintang) which are located in the head and belong to the Du meridian to regulate the brain and calm the spirit, choose HT7 (Shenmen) points belonging to the heart meridian to calm the mind and take the intersection point of the three Yin meridians of foot to reconcile Yin and Yang. Considering that the primary pathogenesis of hypertension is deficiency of kidney Yin and hyperactivity of liver Yang, LR3 (Taichong) point of liver meridian is added to tranquilizing liver Yang and KI3 (Taixi) point of kidney meridian to nourish kidney Yin. According to the theory of TCM and some previous studies, acupuncture at the above points can solve the related symptoms of insomnia (48) and reduce blood pressure (49).

However, there are still some limitations in this study. (1) There is no follow-up in this study due to the limited conditions. However, it is necessary to observe the long-term effects of acupuncture on regulating blood pressure and improving sleep quality because most patients have a long course of disease and are difficult to cure, whether it is hypertension or insomnia. (2) The single-center experimental design will result in a single sample, with limited representativeness, and possible experimental bias. More influencing factors should be considered and the result should be further verified and explored in a large sample population. (3) The patients included in this study ranged from 18 to 70 years old, which might affect the results owing to the hypertension and insomnia rates of the elderly were higher than those of the young. Therefore, age stratification and subgroup analysis will be conducted during the result analysis. (4) The blood pressure data collection in this study is home self-measured blood pressure. Due to the limitation of the subject equipment, the 24-h ambulatory blood pressure changes of the patients, especially the nighttime blood pressure changes, could not be collected. But studies have shown that blood pressure indicators associated with increased risk of cardiovascular disease are nighttime, 24 h, daytime, home self-assessment, and office blood pressure in order of correlation (50). And there is currently evidence to support (51) insomnia as a risk factor for nocturnal hypertension and abnormal blood pressure rhythms. Therefore, it is necessary to pay attention to the blood pressure during sleep in patients with hypertension and insomnia. In the future, smart wearable devices that can regularly monitor patients' 24-h blood pressure and sleep can be used to further investigate the effect of acupuncture on hypertension with insomnia.

## Ethics statement

The studies involving human participants were reviewed and approved by Ethics Committee of Jiangsu Provincial Hospital of Traditional Chinese Medicine. The patients/participants provided their written informed consent to participate in this study.

## Author contributions

WW, PW, and XW conceived of this experiment and participated in experimental design. PW and XW are responsible for drafting the manuscript. CL, SQ, QW, and SL modified the original manuscript. WW and PW are the supervisors of this study. All authors read this manuscript and approved the publication of this protocol.
